# Biomarkers Predicting Poor Prognosis in Covid-19 Patients: A Survival Analysis

**DOI:** 10.7759/cureus.33921

**Published:** 2023-01-18

**Authors:** Amjad Idrissi, Asmae Lekfif, Abdessamad Amrani, Abdelkader Yacoubi, Abir Yahyaoui, Sabrina Belmahi, Oumaima Nassiri, Imane Elmezgueldi, El-Houcine Sebbar, Mohammed Choukri

**Affiliations:** 1 Laboratory of Biochemistry, Central Laboratory Department, Mohammed VI University Hospital, Faculty of Medicine of Oujda, Mohammed First University, Oujda, MAR; 2 Epidemiology, Clinical Research, and Public Health Department, Mohammed VI University Hospital, Faculty of Medicine of Oujda, Mohammed First University, Oujda, MAR; 3 Public Health Department, Regional Administration of Health and Social Protection - Eastern Region, Moroccan Ministry of Health, Oujda, MAR

**Keywords:** survival, retrospective cohort study, prognostic factors, biological parameters, covid-19

## Abstract

Introduction

With the spread of the Covid-19 pandemic and its overwhelming impact on health systems in several countries, the importance of identifying predictors of severity is of paramount importance. The objective of this study is to determine the relationship between death and the biological parameters of patients with Covid-19.

Materials and methods

This is an analytical retrospective cohort study conducted on 326 patients admitted to the Mohammed VI University Hospital in Oujda, Morocco. The statistical analysis concerned the biological parameters carried out on the admission of the patients, in addition to age and sex. The comparison between the two surviving and non-surviving groups was made by a simple analysis than a multivariate analysis by logistic regression. Next, a survival analysis was performed by the Kaplan-Meier method and then by Cox regression.

Results

A total of 326 patients were included in the study, including 108 fatal cases. The mean age was 64.66 ± 15.51 and the sex ratio was 1.08:1 (M:F). Age, procalcitonin, liver enzymes, and coagulation factors were significantly higher in patients who died of Covid-19 and are therefore considered to be the main prognostic factors identified in this study.

Conclusion

Knowledge and monitoring of predictive biomarkers of poor prognosis in patients with Covid-19 could be of great help in the identification of patients at risk and in the implementation of an effective diagnostic and therapeutic strategy to predict severe disease forms.

## Introduction

At the end of December 2019, an atypical pneumonia of unknown etiology appeared in Wuhan, China. The responsible virus which was later identified as a new beta Coronavirus with enveloped ribonucleic acid (RNA), officially called severe acute respiratory syndrome coronavirus 2 (SARS-CoV-2), while the disease it caused was named Coronavirus disease or Covid-19 [1]. Although Covid-19 is considered a lung disease, it has a wide organotropism and can have neurological, nephrological, cardiological, and hematological manifestations among others [2,3]. Covid-19 rapidly spread worldwide, prompting WHO to officially declare it a pandemic on March 11, 2020 [4]. In Morocco, the first case of Covid-19 appeared on March 2, 2020, before it spread over the majority of Moroccan regions [5]. The disease has symptoms with varying degrees of severity: whereas most patients had a favorable clinical course, others were completely asymptomatic. Still, a significant proportion of patients developed severe and life-threatening clinical situations requiring transfer to intensive care units (ICU) [6,7].

The understanding of the clinical, biological, and radiological manifestations of SARS-CoV-2 infection progressed in parallel with the rapid publication of Covid-19 studies including those investigating the correlation between changes in biological parameters and patient prognosis [8]. Thus, a better knowledge of prognostic biomarkers will allow a better allocation of resources, especially when the capacity of health systems including ICUs gets overwhelmed during the pandemic peaks.

The main objective of this study is to assess biomarkers associated with the severity of the disease in patients with Covid-19 to improve the timing of clinical and paraclinical interventions by early identifying patients likely to have a rapidly unfavorable course.

## Materials and methods

This is an analytical retrospective cohort study conducted on 326 patients, aged 18 years or older, infected with SARS-CoV-2, who were hospitalized at Mohammed VI University Hospital in Oujda over a four-month period, from 30/07/2021 to 01/11/2021. The infection with Covid-19 was confirmed by the search for the viral RNA - at the central laboratory of the Mohammed VI University Hospital, in line with international standards using the reverse-transcription polymerase chain reaction (RT-PCR) technique - on nasopharyngeal samples from all patients having presented clinical, biological, and/or radiological symptoms of infection with SARS-CoV-2. The patients were hospitalized according to the severity of the infection either in an ICU or in other non-ICUs. They all benefited from at least one biological assessment per day during their hospitalization, and all these assessments were carried out at the central laboratory of Mohammed VI University Hospital. The biochemical parameters studied were verified - according to the verification protocol of assay methods (scope A) - and by comparing two analyzers: The value of procalcitonin (PCT) was checked and compared in both Abbott's two Architect ci8200 and ROCHE's Cobas E411. The results obtained allowed us to verify the performance of the used assay methods and to compare them with the analytical objectives set in the accreditation process - in accordance with the ISO-15189 standard - to which our laboratory is committed.

The analyzed results were those obtained at the admission of patients of our series. The biological parameter values have been interpreted through the Statistical Product and Service Solutions (SPSS) (IBM SPSS Statistics for Windows, Version 25.0, Armonk, NY) software as qualitative variables (high, normal, or low) according to each biological parameter physiological threshold.

Data was archived in Excel and then exported to IBM SPSS, used for all statistical analyses. All the variables were then expressed in numbers and percentages except for age and length of hospital stay which were expressed as a mean ± standard deviation. Initially, a statistical analysis was carried out by comparing the variables in the two groups of patients: discharged and deceased, Pearson chi-square test or Fisher exact test, and then a multivariate analysis by a binary logistic regression was carried out. Only the variables with a statistically significant test to the univariate analysis were considered relevant for hospital mortality and therefore selected for the model using a step-by-step bottom-up method. Secondarily, a survival analysis was first done using the Kaplan-Meier method and then Cox regression. Factors with a significant Log Rank test were included in the model by always the step-by-step bottom-up method to avoid an over-adjustment in the model. The time delay prior to the event (death) was measured in days from the admission date to the hospital to the date of death in the hospital. Measures of association (odds ratios (OR) and hazard ratios (HR)) were reported with their 95% confidence intervals. The final models of the multivariate analyses (logistic regression and Cox regression) present only those variables with a statistically significant association: a value of p≤0.05 was considered statistically significant for all the analysis results.

## Results

Descriptive analysis

A total of 326 patients were included in the study. The mean age was 64.66 ± 15.51 years. Overall, 181 of them were males (55.5%) and the other 145 were females (44.5%). One hundred eight patients died due to Covid-19-related illness with a case fatality rate of 33.12% in the total sample. A total of 246 patients (75.5%) were hospitalized in an ICU, and 80 patients (24.5%) were hospitalized in other non-ICUs, four of which died (1.23%). The incidence of mortality was 33.12% in the total sample and 31.10% in critically ill patients (admitted to the ICU). The results of the biological assays showed that most patients had high levels of ferritin (89.60%), C reactive protein (CRP) (98.10%), lactic dehydrogenase (LDH) (96.10%), aspartate aminotransferase (AST) (69.60%), blood glucose (66.70%), D-dimers (87.30%), and fibrinogen (83.50%). We also observed the predominance of lymphopenia (64.60%) and hypoalbuminemia (62.50%) in patients who tested positive for SARS-CoV-2. These frequencies are even higher in deceased patients when compared with those who survived.

Statistical analysis

We investigated the association between biological parameters and death. The results of the univariate analysis are shown in Table 1.

**Table 1:** Results of assays and analysis of biomarkers in patients of our series from July 30, 2021 to November 1, 2021 * p ≤0.05 is considered statistically significant, M: Male, F: Female, PCT: procalcitonin, LDH: lactic dehydrogenase, CRP: C reactive protein, AST: aspartate aminotransferase, ALT: alanine transaminase, GGT: gamma-glutamyl transferase, ALP: alkaline phosphatase, PT: prothrombin time

Biomarkers	Values	Total sample workforce (%)	Deceased: number (%)	Survivors: staff (%)	Chi-two test results (p)
PCT	Physiological values: <0.2ng/l	155 (47.00)	37 (33.60)	117 (54.20)	0.001*
	High values	175 (53.00)	73 (66.40)	99 (45.80)	
Ferritin	Physiological values: 20 to 200 μg/l	29 (9.10)	6 (5.70)	23 (11.10)	0.137
	Hypoferritinemia	4 (1.30)	1 (0.90)	3 (1.40)	
	Hyperferritinemia	284 (89.60)	99 (93.40)	182 (87.50)	
Troponin	Physiological values: <26ng/l	180 (59.60)	48 (46.60)	131 (66.8)	0.001*
	High values	122 (40.40)	55 (53.40)	65 (33.20)	
CRP	Physiological values: <5mg/l	6 (1.90)	3 (2.70)	3 (1.40)	0.555
	High values	318 (98.10)	107 (97.30)	208 (98.60)	
LDH	Physiological values: 125 to 243 IU/L	12 (3.90)	1 (1.00)	11 (5.40)	0.045*
	High	298 (96.10)	104 (99.00)	191 (94.60)	
AST	Physiological values: 5 to 34 IU/L	93 (30.40)	23 (22.10)	70 (35.20)	0.013*
	High	213 (69.60)	81 (77.90)	129 (64.80)	
ALT	Physiological values: 0 to 55 IU/L	244 (79.50)	79 (76.00)	163 (81.50)	0.238
	High	63 (20.50)	25 (24.00)	37 (18.50)	
Serum creatinine	Normal: (M: 7.2 to 12.5, F: 5.7 to 11.1 mg/l)	192 (60.00)	53 (48.60)	137 (65.60)	0.001*
	Hypocreatininemia	34 (10.60)	10 (9.20)	24 (11.50)	
	Hypercreatininemia	94 (29.40)	46 (42.20)	48 (23.00)	
Uraemia	Physiological values: 0.15 to 0.45 g/l	162 (50.00)	45 (40.90)	116 (54.70)	0.004*
	Low values	8 (2.50)	1 (0.90)	7 (3.30)	
	High values	154 (47.50)	64 (58.20)	89 (42.00)	
Glycemia	Normal: 0.60 to 1.10 g/l	99 (30.60)	30 (27.50)	69 (32.50)	0.197
	Hypoglycaemia	9 (2.80)	2 (1.80)	7 (3.30)	
	Hyperglycaemia	216 (66.70)	77 (70.60)	136 (64.20)	
GGT	Normal: (M: 12-64, F: 9-36 IU/L)	128 (43.50)	39 (38.60)	88 (46.30)	0.207
	High	166 (56.50)	62 (61.40)	102 (53.70)	
ALP	Physiological values: 40 to 150 IU/L	249 (84.70)	87 (87.00)	159 (83.20)	0.330
	Low	17 (5.80)	3 (3.00)	14 (7.30)	
	High	28 (9.50)	10 (10.00)	18 (9.40)	
Total bilirubin	Normal: 2 to 12 mg/l	249 (92.60)	80 (87.00)	166 (95.40)	0.013*
	High	20 (7.40)	12 (13.00)	8 (4.60)	
Kalemia	Normal: 3.50 to 5.10 mEq/L	237 (72.90)	85 (77.30)	150 (70.80)	0.462
	Hypokalemia	58 (17.80)	17 (15.50)	40 (18.90)	
	Hyperkalemia	30 (9.20)	8 (7.30)	22 (10.40)	
Natremia	Normal: 136 to 145 mEq/L	167 (51.20)	49 (44.50)	117 (54.90)	0.010*
	Hyponatremia	141 (43.30)	50 (45.50)	90 (42.30)	
	Hypernatremia	18 (5.50)	11 (10.00)	6 (2.80)	
Chloremia	Normal: 98 to 107 mEq/L	178 (54.60)	57 (51.80)	121 (56.80)	0.510
	Hypochloremia	122 (37.40)	42 (38.20)	78 (36.60)	
	Hyperchloremia	26 (8.00)	11 (10.00)	14 (6.60)	
Serum calcium	Normal: (84-102 mg/l; Age>60 years: 88-100 mg/l)	135 (41.80)	37 (33.90)	98 (46.40)	0.010*
	Hypocalcemia	178 (55.10)	70 (64.20)	105 (49.80)	
	Hypercalcemia	10 (3.10)	2 (1.80)	8 (3.80)	
Albuminemia	Normal: 35 to 50 g/L	120 (37.50)	20 (18.30)	100 (48.10)	<0.0001*
	Hypoalbuminemia	200 (62.50)	89 (81.70)	108 (51.90)	
Protidemia	Normal: Subject bedridden 60-78 g/l; Age>60 years: 58-76 g/l	254 (78.90)	89 (81.70)	162 (77.10)	0.608
	Hypoprotidemia	34 (10.60)	11 (10.10)	23 (11.00)	
	Hyperprotidemia	34 (10.60)	9(8.30)	25 (11.90)	
Lymphocytes	Physiological rate: 1000.00 to 4000.00/μl	110 (33.80)	31 (28.40)	79 (37.10)	0.183
	Lymphopenia	210 (64.60)	75 (68.80)	132 (62.00)	
	Hyperlymphocytosis	5 (1.50)	3 (2.80)	2 (0.90)	
White blood cells	Physiological rate: 4 000.00-10 000.00/μl	144 (44.30)	38 (34.90)	105 (49.30)	0.015*
	Leukopenia	21 (6.50)	5 (4.60)	16 (7.50)	
	Hyperleukocytosis	160 (49.20)	66 (60.60)	92 (43.20)	
Platelets	Physiological rate: 150 000.00-400 000.00/μl	221 (68.00)	76 (70.40)	142 (66.70)	0.129
	Thrombocytopenia	73 (22.50)	18 (16.70)	54 (25.40)	
	Thrombocytosis	31 (9.50)	14 (13.00)	17 (8.00)	
Prothrombin (PT) levels	Normal: 70 to 100%	250 (79.10)	73 (67.60)	175 (85.40)	<0.0001*
	Low	66 (20.90)	35 (32.40)	30 (14.60)	
D-dimers	Physiological Values: <0.50 mg	42 (12.70)	6 (5.50)	36 (16.70)	0.005*
	High	288 (87.30)	104 (94.50)	180 (83.30)	
Fibrinogen	Physiological rate: 2 to 4 g/l	52 (16.60)	11 (10.50)	40 (19.50)	0.042*
	High	261 (83.40)	94 (89.50)	165 (80.50)	

After analyzing the results, we did not find a statistically significant association between sex and death (p=0.272). We observed that patients over the age of 65 had a higher fatality rate compared to those who were <65 years, with a significant difference (41.8 vs. 25.9%; p=0.003).

The biomarkers that are statistically associated with death are PCT, troponin, LDH, AST, creatinine, urea, total bilirubin (BT), natremia, serum calcium, albuminemia, white blood cell count, prothrombin time (PT), D-dimers, and fibrinogen levels (Table 1).

Multivariate analysis revealed a statistically significant association between death and high levels of PCT, BT, and fibrinogen, and between death and hypoalbuminemia (Table 2).

**Table 2:** Biological parameters statistically associated with death in Covid-19 patients: results of binary logistic regression analysis OR: odds ratio, CI: confidence interval

Variables	OR	95% CI	P
Procalcitonin	1.78	1.01-3.14	0.046
Total bilirubin	3.05	1.12-8.29	0.029
Albumin	4.47	2.32-8.59	<0.0001
Fibrinogen	3.66	1.46-9.13	0.006

Survival analysis

Among 326 patients hospitalized at Mohammed VI University Hospital in Oujda during the study period, we counted 108 deaths (33.12%). The average length of stay was 10.95 ± 7.81 days, and the median observation period was 21 days (IQR 39-10) (Table 3).

**Table 3:** Univariate survival analysis by Kaplan Meier's method of age, sex, and biomarkers of Covid-19 patients in our series Median survival**: the value of ti for which S(ti) = 0.5: Probability of living being = 50%; IQR: the value of ti for which S(ti) = 0.75 and S(ti) = 0.25 respectively. (.. ***): Median survival or IQR is not available: ti could not be calculated because we did not observe a percentage of 50% or 75% of deaths. IQR: Interquartile range * p ≤0.05 is considered statistically significant, PCT: procalcitonin, LDH: lactic dehydrogenase, CRP: C reactive protein, AST: aspartate aminotransferase, ALT: alanine transaminase, GGT: gamma-glutamyl transferase, ALP: alkaline phosphatase, PT: prothrombin time

Variables	Values	Staff	Number of deaths	Median survival ** (IQR)	Log Rank test (p)
Sex	Males	167	61	19 (11 - .. ***)	0.633
	Females	154	47	23 (9 - 35)	
Age	Age ≤ 65	165	43	35 (15 - 39)	0.001*
	Age > 65	153	64	17 (8 - 27)	
PCT	Standard rate	152	37	23 (15-39)	<0.0001*
	High	169	71	18 (6-35)	
Troponin	Normal	177	48	23 (13-35)	0.001*
	High	119	55	16 (7-39)	
Ferritinemia	Normal	29	6	.. (19 - .. )	0.583
	Low	3	1	27 (27-27)	
	High	279	98	21 (10-39)	
CRP	Normal	5	2	16 (16-39)	0.707
	High	311	106	21 (10 - .. )	
LDH	Normal	12	1	39 (39-39)	0.084
	High	292	103	20 (10 - .. )	
AST	Normal	92	22	35 (14 - 39)	0.003*
	High	206	80	18 (9 - .. )	
ALT	Normal	238	77	21 (10-39)	0.476
	High	61	25	19 (11 - .. )	
Serum creatinine	Normal	190	53	21 (13 - 35)	0.002*
	Low	30	8	24 (18 - .. )	
	High	93	46	13 (6 - 39)	
Urea	Normal	165	44	21 (14 - .. )	0.003*
	High	152	64	16 (7 - 35)	
Glycemia	Normal	96	28	21 (13 -.. )	0.430
	Hypoglycaemia	8	2	.. (9 - .. )	
	Hyperglycaemia	212	77	21 (9-39)	
GGT	Normal	127	39	20 (12 -.. )	0.541
	High	163	62	21 (9-3 9)	
PAL	Normal	243	86	21 (9-3 9)	0.454
	Low	17	3	20 (13 - 20)	
	High	28	10	16 (12-.. )	
Total bilirubin	Normal	246	80	21 (12-39)	0.002*
	High	19	12	11 (6-16)	
Kalemia	Normal	231	83	20 (11 -39)	0.998
	Hypokalemia	57	17	21 (9 -.. )	
	Hyperkalemia	29	8	27 (12-27)	
Natremia	Normal	161	47	24 (12-.. )	0.020*
	Low	140	50	18 (10-39)	
	High	17	11	12 (7-12)	
Chloremia	Normal	176	56	23 (12-39)	0.051
	Low	119	42	20 (9-.. )	
	High	23	10	13 (7- 16)	
Serum calcium	Normal	131	35	35 (14-39)	0.012*
	Hypocalcemia	175	70	18 (8-.. )	
	Hypercalcemia	9	2	23 (23-23)	
Albuminemia	Normal	118	19	35 (19-.. )	<0.0001*
	Hypoalbuminemia	195	88	16 (8-25)	
Protidemia	Normal	247	87	19 (9 - .. )	0.504
	Hypoprotidemia	33	11	21 (10-.. )	
	Hyperprotidemia	34	9	39 (10-39)	
Lymphocyte count	Normal	110	32	23 (12-39)	0.415
	Lymphopenia	207	75	20 (10 - .. )	
White blood cells	Normal	143	38	20 (12-35)	0.309
	Leukopenia	21	5	20 (12-21)	
	Hyperleukocytosis	153	64	21 (7-39)	
Platelet count	Normal	214	74	20 (11-39)	0.244
	Thrombocytopenia	71	18	24 (10-.. )	
	Thrombocytosis	31	14	16 (9-21)	
Prothrombin (PT) levels	Normal	247	73	22 (11-.. )	0.004*
	Low	61	33	13 (27-6)	
Fibrinogen	Normal	50	11	39 (10-39)	0.504
	High	259	94	20 (10-.. )	
D-Dimers	Normal	42	6	39 (25-39)	0.007*
	High	279	102	19 (9-.. )	

Based on these results (Table 3), it can be deduced that patients aged less than 65 years had higher median survival than those aged over 65 years (35 vs. 17 days; p=0.001). The same is true for patients with normal levels of PCT, troponin, AST, creatinine, urea, BT, natremia, chloremia, and serum calcium, with a statistically significant difference. We also noted that the occurrence of death was earlier in patients with hypoalbuminemia (median survival: 35 vs. 16 days; p<0.0001); low prothrombin levels (22 vs. 13 days; p=0.008), and high D-dimer levels (39 vs. 19 days; p=0.011).

In multivariate analysis, we observed that old age (>65 years), high levels of PCT, and disruption of liver function (hypoalbuminemia and elevated AST and BT) are factors that significantly reduce the survival time of patients hospitalized for SARS-CoV-2 infection (Table 4).

**Table 4:** Factors statistically associated with death in Covid-19 patients of our series, after doing a multivariate analysis using the Cox model AST: aspartate aminotransferase, PCT: procalcitonin, CI: confidence interval

Explanatory variables	Hazard ratio	95% CI	Meaning (p)
AGE	1.61	1.03-2.52	0.036
PCT	2.07	1.31-3.27	0.002
Albuminemia	2.82	1.56-5.07	0.001
Total bilirubin	1.99	1.07-3.71	0.030
AST	1.67	1.00-2.78	0.050

**Figure 1:**
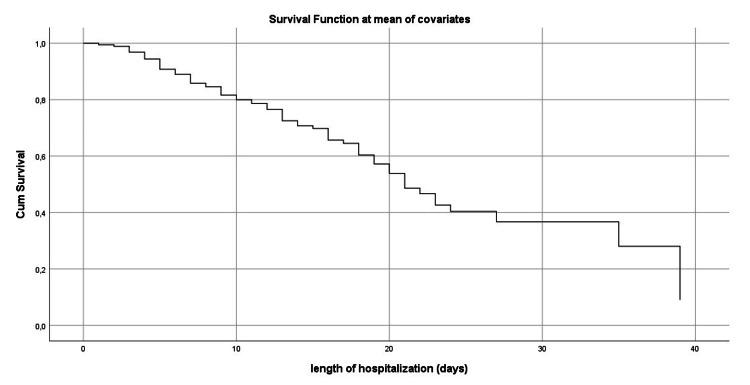
Cumulative survival curve (final cox regression model)

**Figure 2:**
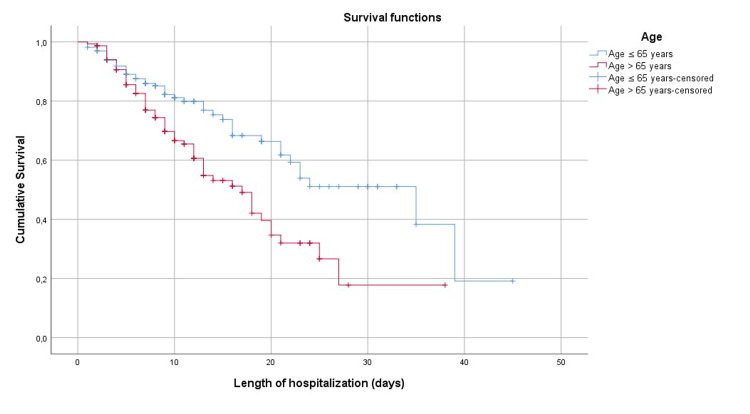
Survival curve by Kaplan Meier's method in Covid-19 patients in our series, according to age

**Figure 3:**
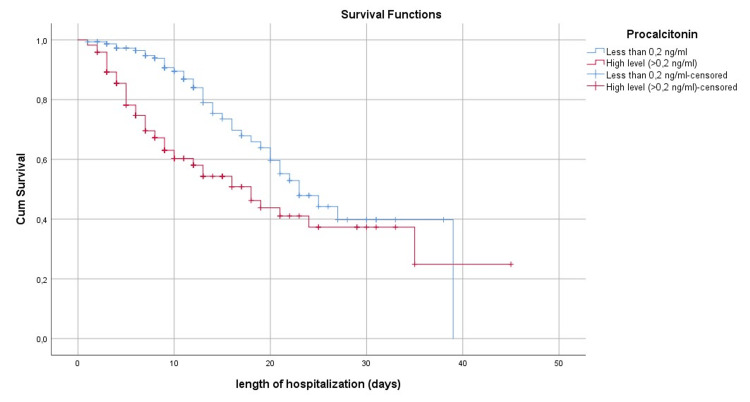
Survival curve by the Kaplan Meier method, in Covid-19 patients of our series according to the procalcitonin dosage

**Figure 4:**
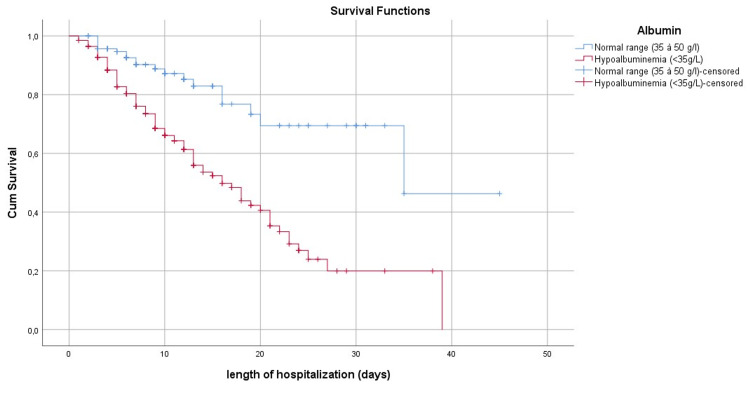
Survival curve by Kaplan Meier's method, in patients with Covid-19 in our series, according to the dosage of albuminemia

**Figure 5:**
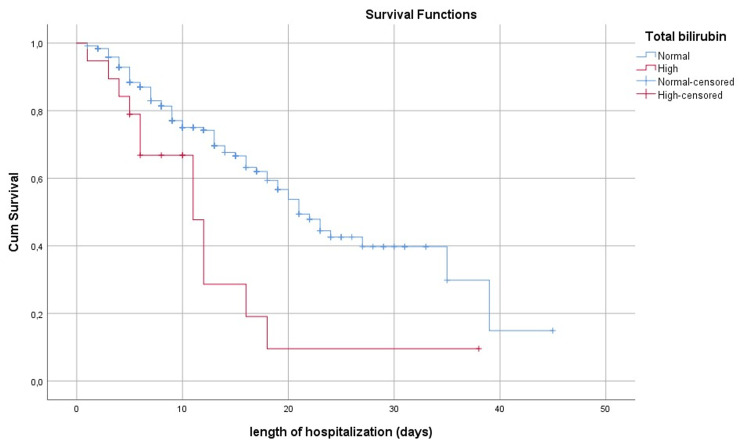
Survival curve by the Kaplan Meier method, in Covid-19 patients in our series according to the dosage of total bilirubin

**Figure 6:**
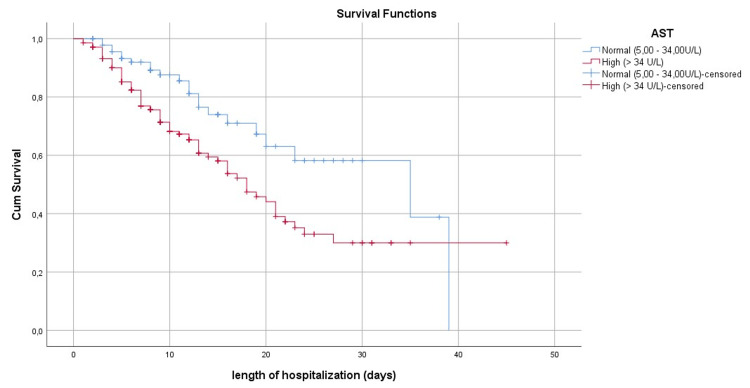
Survival curve by the Kaplan Meier method, in patients with Covid-19 of our series, according to the assay of aspartate aminotransferase (AST)

Figure 1 shows the survival curve at the mean of the Co variables while Figures 2-6 show, respectively, the survival curves - using the Kaplan Meier method - as a function of age, PCT, albuminemia, BT, and AST.

## Discussion

The worldwide spread of Coronavirus infection and the daily communication of the number of new cases, especially death numbers, have drawn the attention of researchers toward the variability of clinical pictures between asymptomatic patients and others developing serious clinical forms sometimes with risk of death. This study was conducted to investigate the relationship between laboratory parameters and the prognosis of Covid-19 patients.

Among 326 patients hospitalized during the four-month study, 108 patients presented the event of death (33.12%). Statistical analysis objectified a significant association between death and high levels of PCT (p=0.046), supporting the findings of other studies that have also identified PCT as one of the highly significant prognostic factors for hospital mortality [9-12]. We also highlighted a statistically significant difference between the two groups of surviving and non-surviving patients in terms of coagulation factors, specifically prothrombin (p<0.0001), D-dimer levels (p=0.005), and fibrinogen levels (p=0.042). Coagulation abnormalities are frequently reported in the case of Covid-19 (what is called coagulopathy associated with Covid-19). It is most often described by an increase in the plasma level of D-dimers. Thrombotic complications including disseminated intravascular coagulation (DIC) are more common complications in non-survivors. In this sense, multiple studies exploring the relationship between increased D-dimer levels and prognosis concluded that the severity of Covid-19 can be assessed by the rate of D-dimers at admission [13-16]. These studies have also revealed, in addition to increased D-dimers, higher levels of fibrinogen and fibrin breakdown products (FDP), longer activated prothrombin time, and partial thromboplastin time at admission, which are common in the event of death in patients with SARS-CoV-2 pneumonia [12,17-19]. Excess of inflammation related to massive cytokine release, endothelial dysfunction, vascular stasis, and platelet activation are phenomena that appear to be entangled in Covid-19-related coagulopathy [20]. We did not find a significant association between leukocyte level and lymphopenia on the one hand and death on the other. However, it should be noted that in other articles, the degree of lymphocytopenia was associated with the severity of the disease and predictive of it. The same is true for the change in white blood cell count which can be considered one of the alarming prognostic factors [11,19,21,22].

If Covid-19 can be manifested in infectious pneumonia, some patients also develop gastrointestinal and hepatic manifestations. The most frequent symptoms are diarrhea, vomiting, and nausea, sometimes accompanied by disturbances in liver balance represented mainly by an increase in the levels of AST, alanine aminotransferase (ALT), BT, and hypoalbuminemia [23,24]. Not only did our study confirm these findings but it also identified increased total bilirubin and AST and low albumin concentrations as prognostic factors for Covid-19, and that patients with higher-than-normal BT levels have a three-fold increased risk of death (OR=3.05 95% CI (1.12-8.29); p=0.029) compared to those with normal BT (p=0.029). Our findings affirm the ones reported by Ding et al., which suggest the monitoring of AST and BT levels in hospitalized patients with Covid-19 [25]. However, in the absence of clinical data and information about the history of our patients, including those concerned by the presence or absence of chronic liver disease, it is difficult to discuss whether such disturbances observed in these patients can be considered as a complication related to Covid-19 or a worsening of underlying chronic liver disease, although the literature has reported that patients with chronic liver disease are more likely to develop a severe and critical clinical picture of Covid-19 [26].

The results of the survival analysis showed an earlier risk of death in patients with high PCT levels and disturbed liver function. This confirms the importance of taking these parameters into account upon admission for more appropriate management with corresponding monitoring of liver function. Patients older than 65 years have an earlier risk of death multiplied by 1.61 compared to other patients. Regardless of other factors, this relationship between age and risk of death has been observed since the beginning of the Covid-19 pandemic as confirmed by several studies [2,12,22,27,28]. The age-specific survival curve (Figure 2) illustrates the difference in cumulative survival between the two groups of patients during the hospitalization period. As for sex, we did not find a significant relationship between sex and death. This result is similar to the studies carried out by Bastug et al. [7] and Kocayiğit et al. [12]; Although other studies have managed to highlight a statistically significant relationship between the male sex and death [11,27].

Our study had some limitations. To begin with, the patients included in the study were all hospitalized patients, who required management in the hospital setting. This suggests that all these patients had a moderate to severe clinical picture since admission. Secondly, there was a lack of clinical and radiological information especially scanographic data, which, in addition to biological analyses, could have helped us assess the severity of the infection attack on these patients. Nevertheless, our main objective was to assess the severity of the SARS-Cov-2 attack based on biomarker results. While the consideration of clinical and scanographic data would have been of great importance, this does not contradict the validity of our results, supported by multivariate analyses and by the fact that most patients were able to benefit from a complete biological assessment. This suggests that the proportion of missing data is low and the statistical power is high.

## Conclusions

At the end of this study, we found that, in addition to age, PCT, liver markers, and coagulation parameters can be considered prognostic factors to be considered when assessing the severity of a Covid-19 attack. Nevertheless, other studies exploring the pathophysiology of these disturbances and identifying the risk factors related to severe forms or lethality caused by Covid-19 are of great importance to better understand the differences in severity observed in these patients. This will develop risk stratification modalities which will in turn optimize the management of these patients.
